# Fit and Retention of Cobalt–Chromium Removable Partial Denture Frameworks Fabricated with Selective Laser Melting

**DOI:** 10.3390/jfb14080416

**Published:** 2023-08-08

**Authors:** Stefan Rues, Akinori Tasaka, Isabella Fleckenstein, Shuichiro Yamashita, Peter Rammelsberg, Sophia Boehm, Franz Sebastian Schwindling

**Affiliations:** 1 Department of Prosthodontics, Heidelberg University Hospital, 69120 Heidelberg, Germany; 2 Department of Removable Prosthodontics, Tokyo Dental College, Tokyo 101-0061, Japan; 3 Department of Prosthetic Dentistry, Medical University Innsbruck, 6020 Innsbruck, Austria

**Keywords:** accuracy, fit, retention, cobalt–chromium, removable partial denture, selective laser melting

## Abstract

Purpose: To evaluate fit and retention of cobalt–chromium removable partial denture (RPD) frameworks fabricated with selective laser melting (SLM). Methods: Three types of framework for clasp-retained RPDs were virtually designed and fabricated using SLM (n = 30). For comparison, 30 additional frameworks were produced using conventional lost-wax casting. A biomechanical model was created, incorporating extracted teeth mounted on flexible metal posts. Using this model, horizontal constraint forces resulting from a misfit were measured using strain gauges, while vertical forces were not recorded. The constraint force components and resultant forces were determined for all abutment teeth, and the maximum retention force during RPD removal from the model was also assessed. For statistical evaluation, the two fabrication methods were analyzed by calculating the means and standard deviations. Results: The average horizontal constraint forces showed similar values for both fabrication methods (SLM: 3.5 ± 1.0 N, casting: 3.4 ± 1.6 N). The overall scatter of data for cast RPDs was greater compared to those fabricated using SLM, indicating a better reproducibility of the SLM process. With regard to retention, the intended retention force of 5–10 N per abutment tooth was not attained in one of the cast groups, while it was consistently achieved in all SLM groups. Conclusions: This in vitro study found that SLM is a promising option for the manufacture of cobalt–chromium RPD frameworks in terms of fit and retention.

## 1. Introduction

The issue of multiple tooth loss remains relevant, affecting a considerable number of individuals, with approximately 9% of adults in the United States experiencing severe tooth loss [1]. To address this clinical problem, clasp-retained removable partial dentures (RPDs) have emerged as a widely employed standard treatment method. When these restorations are of high quality, they hold the potential to significantly improve patient satisfaction [2]. However, it is important to note that clasp-retained RPDs often require maintenance due to complications [3,4]. In this context, technological advancements could alleviate some of the limitations associated with current approaches [5]. More specifically, the additive fabrication technique of selective laser melting (SLM) has been proposed for manufacturing RPD frameworks [6]. In SLM, metallic powders are fused together with a laser beam. Accurate fit and adequate retention are prerequisites for the use of SLM-fabricated frameworks in clinical routine.

When it comes to the accuracy of fabrication, the existing evidence presents a contradictory picture, with findings ranging from statements declaring it “unsuitable for clinical use” [7] to claims that it is “more accurate than the conventional technique” [8]. These divergent findings may highlight the sensitivity of SLM-production to various factors, including powder composition, layer thickness, nesting orientation, or model support [9]. In addition to refining the SLM process itself, operators can enhance accuracy by connecting clasp tips to create closed rings or by employing heat treatment to reduce residual stresses [10]. These measures are essential in achieving the accuracy required for clinical application in dentistry.

Ensuring the proper fit of clasp-retained RPDs is crucial, but an adequate retention force during their removal is similarly important. The retention force at each abutment tooth should strike a balance: it should be sufficiently high to securely maintain the RPD in its intended position, yet low enough to prevent any adverse effects on the periodontal ligament of the abutment teeth. Typically, a maximum retentive force ranging between 5 and 10 N has been recommended for various types of attachments [11,12,13,14].

The objective of this study was to conduct a comprehensive examination of the two crucial factors, namely fit and retention, in clasp-retained RPDs fabricated through conventional casting and with SLM. Therefore, a metal-based model with natural teeth was designed. This model simulated the horizontal resilience of abutment teeth, as previously described [15]. The constraint forces exerted on each individual abutment tooth after integrating the RPD could be measured by means of strain gauges. These forces directly indicate fabrication inaccuracies, as horizontal constraint forces are a consequence of horizontal tooth movement caused by ill-fitting RPDs. High misfit-induced forces can negatively impact patient comfort [16] and should be diligently minimized. Another important feature of the present test setup is the fact that the maximum retention force during the removal of the RPDs was determined immediately after having recorded fit. This approach allowed one to assess the implications of fabrication inaccuracies on retention.

The null hypotheses of this study were that SLM-fabricated cobalt–chromium RPD frameworks and frameworks fabricated using the lost-wax casting method would not differ with regard to two aspects: (i) RPD accuracy, which was assessed by measuring misfit-induced horizontal constraint forces, and (ii) RPD retention, determined by measuring the maximum retention force divided by the respective number of abutment teeth during RPD removal.

## 2. Materials and Methods

### 2.1. Test Model

**Figure 1:**
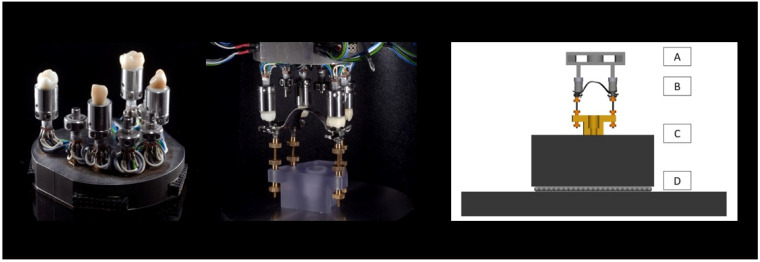
(**Left**): The biomechanical model used to test fit. The model permitted evaluation of different abutment tooth configurations. Middle: The model was mounted upside down for testing in a universal testing machine. The setup allowed vertical movement of the model, while the frameworks were fixed in one place. (**Right**): Schematic of test setup. A: Test model mounted upside down. The model could be moved in vertical direction within the universal testing machine. B: RPD. C: The RPD was fixed to a weight (m = 9 kg), which prevented upward movement of the denture when the test model was moved upward. D: Ball bearing, which allowed horizontal movement of the RPD and weight. RPD, removable partial denture.

A model was produced with stainless-steel posts (1.4521, yield stress > 320 MPa, cross-section 4 × 4 mm) in the positions of teeth 17, 15, 13, 23, 25, and 27. Extracted teeth were attached to these posts. The teeth could be removed from the model by loosening a lock screw and removing a conical splint. To enable measurement of misfit-induced strains caused by deflections of the posts, strain gauges (1-LY13-0.6/120, HBK; Darmstadt, Germany) were attached (Figure 1). The two opposing gauges at each post were connected as half-bridges, which allowed horizontal forces to be amplified and recorded in both the anterior–posterior and lateral–medial directions (QuantumX MX840B, Catman Easy, HBK; Darmstadt, Germany). In advance of this study, strain gauge sensors were calibrated in a universal testing device with calibration forces ranging from −100 N to +100 N in anterior, lateral, and vertical direction and a calibration force increment of 20 N. With the calibration process finished, forces could be calculated based on the measured voltages and measurement errors were determined to be <5%.

**Figure 2:**
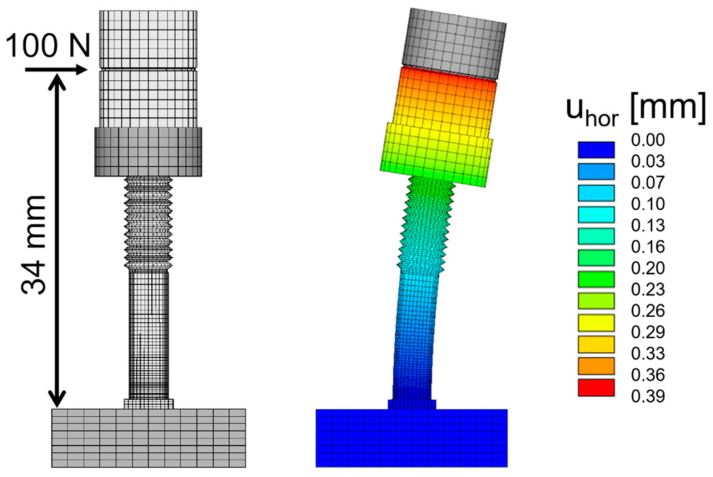
Finite Element model of stainless-steel post welded to the base plate. The calibration abutment possessed a notch at a 34 mm distance from the base plate (**left**) for standardized load application. With a horizontal force of 100 N, the respective displacement at the same height was u_hor_ = 0.39 mm (**right**).

The horizontal resilience of the metal posts welded in the baseplate with respect to horizontal forces acting at tooth-level could be determined with simple mechanical models or finite element analyses (Figure 2). For stainless steel (E = 200 GPa, ν = 0.3), a horizontal force of 100 N was applied to a finite element model (ANSYS R2022 R2, CADFEM; Canonsburg, PA, USA) at the calibration height 34 mm (identical to the height of the undercut regions of the natural teeth) above the base plate caused a deflection of the beam of u_hor_ = 0.39 mm which is in the range of natural tooth mobility [17]. It should be mentioned that with a quadratic cross section the resilience calculated for one horizontal direction is the same in any other horizontal direction. It could also be verified that test forces up to 100 N acting on a single post would not lead to any unwished plastic deformations.

The metal model could be fixed in a universal testing device (Z005, Zwick/Roell; Ulm, Germany) in an upside-down configuration, permitting vertical movement. The direction of this movement coincided with the direction of insertion of the respective RPD onto the abutment teeth.

### 2.2. Framework Types

**Figure 3:**
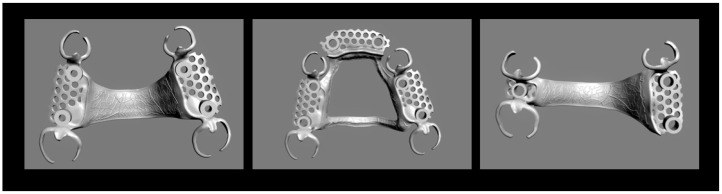
Three types of framework were tested (left to right: A, B, C). Two different abutment tooth configurations were selected: a Kennedy class III configuration (**left** and **middle**) and a Kennedy class II configuration (**right**). For types A and B, the abutment teeth were 17, 13, 23, and 27. The main connector design in framework types A and C (palatal strap) differed from that in type B (anteroposterior bar). For type C, the abutment teeth were 17, 15, and 25.

**Figure 4:**
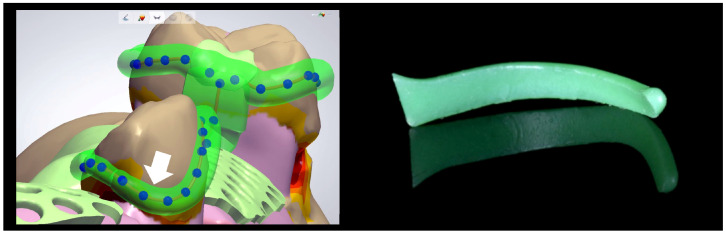
The removable partial denture frameworks were designed using standard dental laboratory software (Dental Designer 2018, 3Shape). (**Left**): The tips of buccal clasp arms were placed in areas with an undercut depth of 0.25 mm (arrow). (**Right**): The shape and cross-section of the clasps were designed to match pre-formed wax patterns for the casting technique.

Two different abutment tooth configurations were selected: a Kennedy class II configuration and a Kennedy class III configuration. High-precision silicone impressions (Flexitime, Kulzer; Wehrheim, Germany) were taken, and one stone master cast was produced for each of the three types of framework: A and B (both Kennedy class III, with two different connector designs; abutment teeth 17, 13, 23, 27; Figure 3, left and middle) and C (Kennedy class II; abutment teeth 17, 15, 23; Figure 3, right). The RPDs were designed as follows: First, the master casts were digitized (D2000, 3Shape; Copenhagen, Denmark) and imported into consumer dental laboratory software (Dental Designer 2018, 3Shape). Undercuts in the direction of insertion were blocked out. In areas where clasps were located, the blocked-out area was trimmed to allow placement of the buccal clasp arms in regions with an undercut depth of 0.25 mm (Figure 4, left) which should result in the aspired maximum clasp retention of 5–10 N during RDP removal. Akers clasps with occlusal rests were chosen in this study. The clasp tips were connected. The longitudinal shape and cross-section of the clasps were designed to match pre-formed wax patterns for the casting technique (premolar and molar clasps, Dentaurum; Ispringen, Germany), which had been digitized and analyzed during pre-trials (Figure 4, right). The design of the main connector in framework types A and C (palatal strap) differed from that in type B (anteroposterior bar). To enable the RPDs to be fixed in place during mechanical testing later on, hollow cylinders were added. These were oriented in the direction of insertion. For SLM fabrication, reinforcement support bars were incorporated.

### 2.3. Framework Production: SLM and Casting

The virtual constructions were sent for SLM fabrication. An EOS SP2 machine and a cobalt–chromium alloy were used (CobaltChrome, EOS, Turku, Finland; 63.8 weight % Co, 24.7% Cr, 5.4% W, 5.1% Mo, 1% Si, <0.5% Fe, <0.1% Mn). A 45° building orientation was selected. A linear support design with additional horizontal structures was used. The laser spot size was 80 µm with a velocity of 300 mm/s (contour) and 762.9 mm/s (infill). After melting and before removal of the support structures, a heat treatment was performed at 1000 °C for 30 min to get rid of residual stresses. The chosen parameters were selected in accordance with the recommendations and expertise of the manufacturer and the producing provider (Infinident; Darmstadt, Germany [18]). To ensure standardization, the frameworks were removed from the building platform and then delivered for testing with no further post-processing. Each framework design was fitted to the respective stone master cast by removing the reinforcement bars, disconnecting the tips of the clasps, removing the excess metal, and finishing the claps.

For casting, the master casts were duplicated with silicone material (Siliform, Dreve; Unna, Germany) after the blocking out of unwanted undercuts. To exactly reproduce the digital framework designs, one of the SLM frameworks was placed on these duplicated models, and its outline was marked using a pen. The pre-formed wax patterns were applied to the model, a tree sprue was added, and the objects were invested with phosphate-bonded precision investment material (Rema dynamic S, Dentaurum; Ispringen, Germany, identical batch number for all samples). The invested wax RPDs were placed in a furnace at room temperature, which was then heated to 950 °C. A vacuum-pressure casting machine (Heracast iQ, Kulzer; Wehrheim, Germany) was used with cobalt–chromium alloy (Remanium GM 800+, Dentaurum; Ispringen, Germany, composition: 58.3 weight % Co, 32.0% Cr, 6.5% Mo, 1.5% W, 1.0% Si, <1% Mn, N, C). Due to the large amount of alloy, the alloy was premelted before placing the preheated mold in the casting device and starting the automated melting and casting process with a casting temperature of 1510 °C. After cooling, the sprues were removed, and the frameworks were fitted to the master cast. To ensure standardization, all dental laboratory steps were performed by the same dental technician. In total, 60 frameworks were produced: 30 by casting and 30 by SLM. For the final RPDs of each design group, clasp end positions of cast or SLM-manufactured restorations were visually inspected and compared.

### 2.4. Fit Tests, RPD Retention, and Statistics

To be able to attach the RPD to the upper part of the testing device, threaded bars were fixated in the cylindrical attachments of the RPDs. Each RPD was first manually fitted to the test model, and the cross-head was lowered until the threaded bars could be connected to a weight placed on a ball bearing using a 3D-printed counterpart. The ball bearing prevented unwanted external horizontal forces or torque with respect to the vertical axis during testing, whereas the weight (m = 9 kg) was heavy enough to test maximum retention during RPD separation without lifting the weight (Figure 1). For each RPD, this first fixation was carried out twice. After fixation, the model was moved upwards until it was completely separated from the teeth. Strain gauge sensors were connected to the amplifier and a zero-force-adjustment took place in this state, thus enabling the measurement of horizontal constraint forces acting.

**Figure 5:**
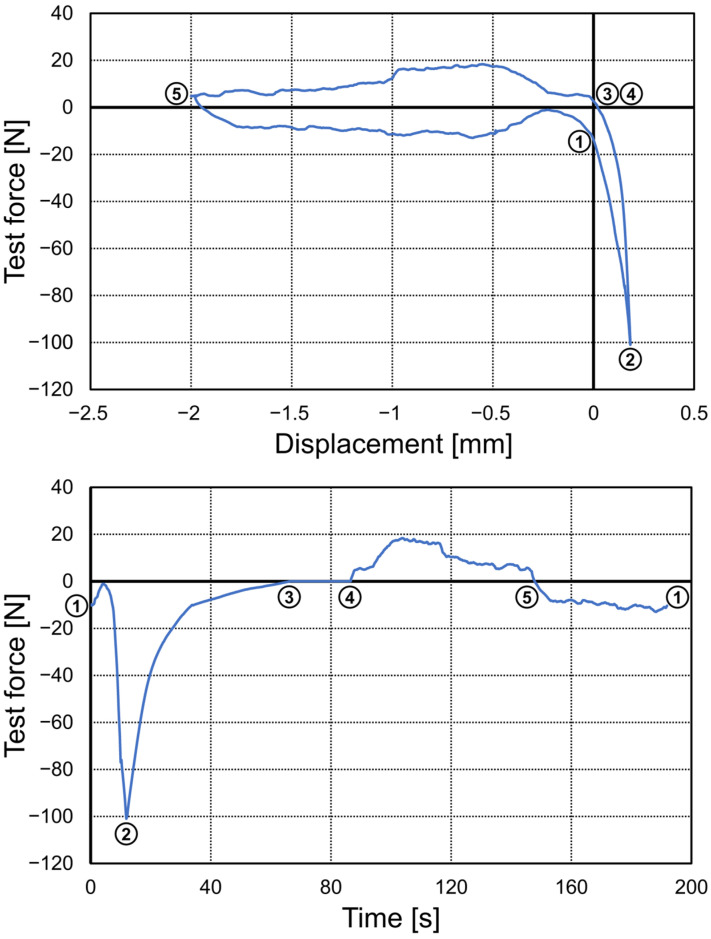
Exemplary force–displacement (**top**) and force–time (**bottom**) diagrams for a representative test cycle in the universal testing device: (1) start position at about 0.2 mm above the final rest position, (2) insertion of the RPD with F = −100 N maximum compressive force, (3) removal of the vertical load, (3)–(4) 20 s period with no vertical load for constraint force measurement, and (5) removal of the RPD with end position 2 mm above the starting position to determine maximum retention. At the end of the cycle, RPD was moved again to the starting position. The upper diagram shows the force–displacement curve whereas the lower diagram shows the force–time.

Beginning from a starting position about 0.2 mm above the final RPD position, automatic test cycles were carried out as given in Figure 5: The insertion process was started, and the test force was increased up to a compressive force of 100 N (crosshead speed of 4 mm/min up to a force of 75 N, then crosshead speed of 1 mm/min). Then, the model with the RPD was moved upwards until the vertical force was equal to 0 ± 0.1 N. This state was kept constant for 20 s (evaluation of constraint forces without vertical load) before maximum retention was tested by moving the model upwards to an end position 2 mm above the starting position which was enough to remove the clasps from the respective undercut regions. In a final step, the crosshead was lowered again to the starting position to be able to start the next test cycle. The model was fitted to the RPD three times. Strain gauge measurements were evaluated for a 5 s period within the 20 s interval with zero vertical force. This procedure was carried out twice since the manual seating of the RPD was repeated (see above). Mean values of all six measurements (two test repetitions with three test cycles each) were averaged. For each abutment tooth (index *i*, *n_abut_*: number of abutment teeth), the measured anterior (*F_ant_*_,*i*_) and lateral (*F_lat_*_,*i*_) force components were used to calculate the resultant horizontal force ($F_{res,i}=\sqrt{F_{ant,i}^{2}+F_{lat,i}^{2}}$) acting on the respective abutment tooth. For each test cycle, an average horizontal constraint force was defined using $F_{res}=\frac{1}{n_{abut}}\sum_{i=1}^{n_{abut}}F_{res,i}$. Force values averaged for all abutment teeth were also calculated for the anterior and lateral force components.

To compare the performance of the framework designs with regard to fit and retention, ANOVAs (factors: Design, Fabrication) and post hoc Tukey tests for the different design groups (SPSS 29, IBM; Armonk, NY, USA) were used at a significance level of α = 0.05. Due to the exploratory nature of the study, all *p*-values must be interpreted descriptively.

### 2.5. Microscopy

To identify differences in the microstructure samples from both casted and SLM-fabricated RPDs, the samples were ground planar and polished (final polishing step with 1 µm diamond grains) and etched for 1 h using 25% hydrochloric acid. SEM (JSM-6510, Jeol; Tokyo, Japan) images were generated with magnifications between 200× and 10,000×.

## 3. Results

The production of identical clasp-retained RPDs using either the casting technique or SLM was successful for RPD design B and C with clasp tip positions in the undercut regions differing less than 0.5 mm when comparing RPDs of the two fabrication types. However, when examining RPDs with design A that were seated in end position on the test model, it was observed that the tips of the clasps on the cast restorations were frequently positioned up to 1 mm higher compared to the SLM-fabricated restorations, indicating limitations of this study, which will be discussed later.

**Figure 6:**
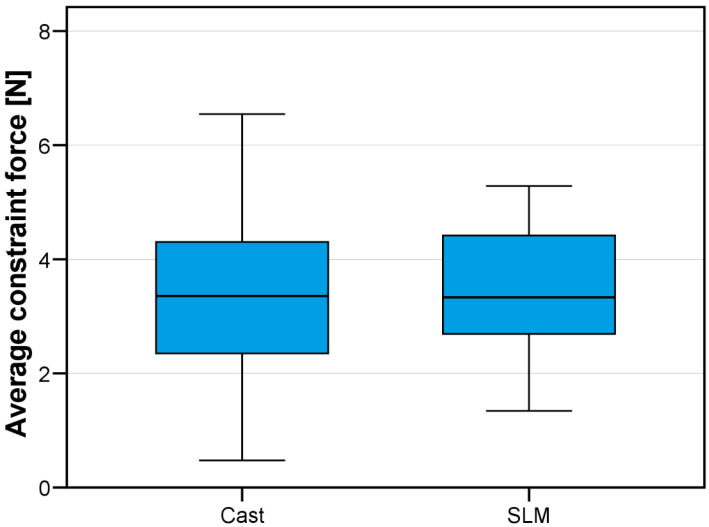
Average horizontal constraint forces in [N] for the study groups. The range of the horizontal constraint forces *F_res_* was smaller in the SLM group than in the cast group, as shown by the shorter boxplot whiskers.

**Figure 7:**
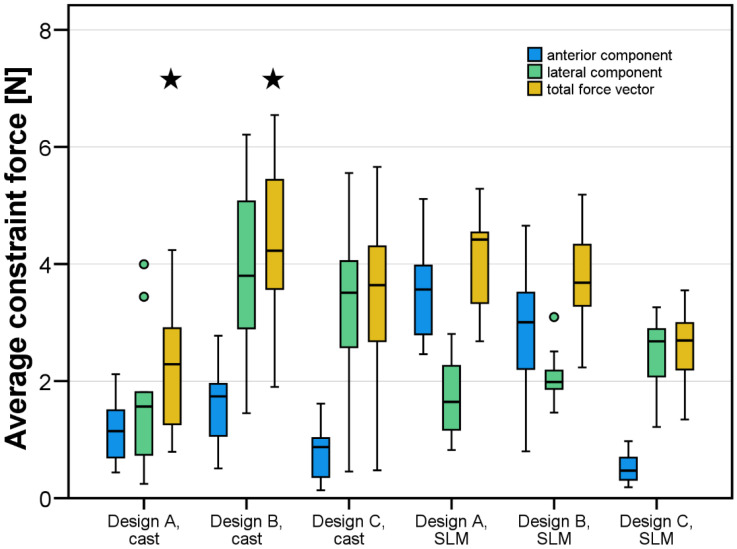
Average anterior, lateral, and total constraint forces in [N]. Significant differences (stars) were only detected within the cast group, between designs A and B.

**Figure 8:**
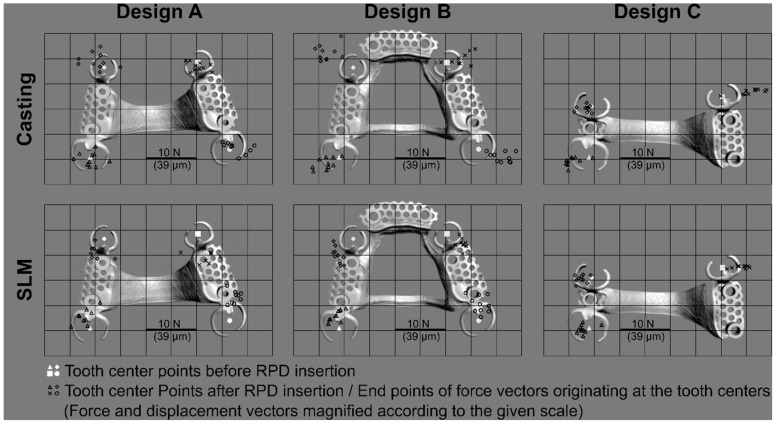
Constraint forces acting on the abutment teeth of each test group (n = 10 for each group) due to insertion of the individual RPD. For RPD geometry and abutment tooth positions before RPD insertion, one grid unit equals 10 mm. The scale for magnified force vectors (or respective displacements vectors) is given in the diagrams.

Overall, average resultant constraint forces were *F_res_* = 3.5 ± 1.0 N for SLM and *F_res_* = 3.4 ± 1.6 N for casting (Figure 6) and thus comparable. Knowing the resilience of the abutment teeth, these forces can be correlated to mean deflections of about 14 µm. The overall scatter of data for cast RPDs was greater compared to additively fabricated RPDs. Maximum forces acting on individual teeth were always below 18 N (or an equivalent deflection of 70 µm). When looking at the data split up in all test groups (Figure 7), ANOVA and Tukey post hoc tests identified the framework design as a significant factor (*p* = 0.014) affecting RPD fit, whereas the fabrication type (*p* = 0.747) was not significant. Since fit of different design groups was differently affected by the fabrication type, the factor combination Design*Type was significant (*p* < 0.001). Design A and C formed one homogeneous subgroup (mean constraint forces *F_res_*_,*mean*_ = 3.16 and *F_res_*_,*mean*_ = 3.08, respectively) whereas Design B showed significantly higher misfit (*F_res_*_,*mean*_ = 4.09, *p* < 0.035 for all pairwise tests). Directional patterns could be analyzed by looking at the measured constraint force components in anterior–posterior and lateral–medial directions. Interestingly, differences were found with regard to force direction between the fabrication techniques. Whereas misfit-induced constraint forces in the SLM groups were predominantly oriented in the anterior–posterior direction, the lateral–medial direction was more pronounced with constraint forces in the cast groups. Constraint forces (or respective horizontal displacements) acting on individual teeth during insertion of all RPDs were visualized in Figure 8. Cast RPDs were in general too large since abutment teeth were pushed away from each other. For SLM-fabricated restorations, on the other hand, RPDs with Design A and Design B were too short in the anterior–posterior direction pulling the teeth towards each other. Here, Design C behaved differently, being in general too large in the lateral–medial direction.

**Figure 9:**
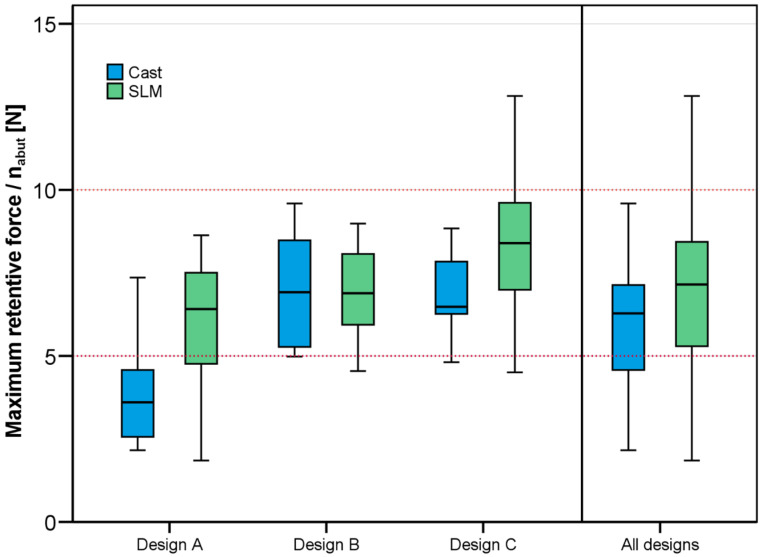
Maximum retentive forces for the three RPD designs and the two manufacturing methods divided by the number of abutment teeth of the respective design (Design A and Design B: *n_abut_* = 4, Design C: *n_abut_* = 3).

The results for maximum retention forces are depicted in Figure 9. Since the number of abutment teeth differed between Design C (*n_abut_* = 3) and the other two designs (*n_abut_* = 4), measured force values were divided by the abutment tooth number. For all test groups except cast RPDs with design A, the targeted value for maximum retention of a single abutment tooth between 5 N and 10 N was well met. Overall, SLM-fabricated RPDs showed slightly higher retention compared to cast RPDs. In detail, both factors, Design (*p* < 0.001) and Type (*p* = 0.010), affected the retention of the RPDs. There were no countercurrent effects (*p* = 0.113 for Design*Type). Design A was associated with significantly lower retentive forces than designs B and C.

**Figure 10:**
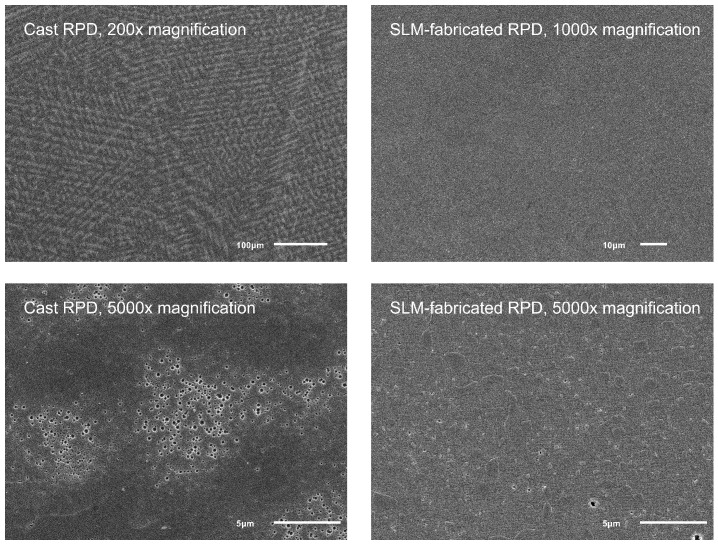
SEM images of polished and etched surfaces of CoCr samples taken from RPDs manufactured with either casting (**left side**) or SLM (**right side**).

When looking at the microstructure of the CoCr alloy (Figure 10) used for casting and SLM, it could be seen that SLM-fabricated CoCr had a finer and more homogeneous microstructure. Furthermore, shrink holes that can never be totally avoided when casting large objects such as RPDs were more frequent than flaws in the microstructure of SLM-fabricated CoCr.

## 4. Discussion

In this study, the overall performance with regard to horizontal fit was comparable for SLM-fabricated RPDs and conventionally cast RPDs (Figure 6). ANOVA did not reveal a significant difference with regard to the type of fabrication. Thus, the null hypothesis with regard to the accuracy of the RPDs could not be rejected. Design B with the most complex geometry (anteroposterior palatal bar) was associated with the highest constraint forces about a third higher than the forces observed for the other groups. In this context, however, results of the cast RPDs with Design A should be interpreted with care since clasp positions were up to 1 mm higher compared to the respective SLM test group. This shows that, even under the conditions of an in vitro study, reproducibility of the RPD geometry was challenging due to many manual and/or sensitive working steps such as generating a duplicate model made of investment material, manually applying wax elements, and performing the lost-wax casting.

It must be clearly stated, however, that because the SLM frameworks were ordered completely unfinished from the SLM provider, extensive work was required to fit them to the model. This is directly linked to the two most important limitations of the study. First, all frameworks were fitted manually, thus introducing the “human factor” to the study setup, even though we tried to standardize the workflow as far as possible (e.g., only one experienced technician performed all the laboratory work). Second, no conclusions on cost-efficiency can be drawn because SLM frameworks are not usually delivered to the dental laboratory in a completely unfinished state.

In the present study, a perfect, passive fit was not achieved for either of the two fabrication methods. It is, however, questionable whether a passive fit (with resulting forces of 0 N) is clinically required. Strain measurements to assess restoration fit have been performed frequently in implant dentistry [19,20,21,22]. These results have shown that passive fit is difficult to achieve and might not be as critical for maintaining osseointegration as expected. For example, Karl and Taylor observed bone adaptation around implants that were restored with non-passively fitting prostheses after 6 months [23], leading to a decrease in strain over time.

The available literature on the fit of SLM frameworks is rather inconsistent. Arnold et al. assessed the fit of SLM-fabricated clasps to abutment teeth by using a light microscope to measure the gaps between them. They concluded that SLM-fabricated samples were substantially less accurate than cast samples [7]. Ye and coworkers evaluated the fit of RPD frameworks in vivo by measuring silicone film thicknesses. The investigation captured local fit but not the overall fit (i.e., the distance between the retentive elements), and it was found that casting performed better than SLM [24]. Soltanzadeh et al. compared gaps between RPD frameworks and a master model [25]. Although both conventional and 3D-printing methods resulted in clinically acceptable adaptation, overall fit and accuracy were better in the conventionally cast RPD groups. Another study found comparable adaptations for SLM-printed and cast RPD frameworks [26]. Even though SLM performed worse than casting in two of four framework types, no significant differences were found regarding the other two configurations. However, the authors conceded that it might be possible to further improve the SLM results by optimizing parameters such as building orientation, support design, laser spot size, laser scan path, and velocity. Bajunaid et al. investigated the fit of RPD frameworks made from SLM and from lost-wax casting. There was a tendency towards SLM performing slightly better [27]. The most favorable results were reported by Forrester et al. in their study of the accuracy of SLM for fabrication of a palatal coverage metal framework. The accuracy of SLM-fabricated frameworks was comparable to that of conventionally cast ones, thus supporting our results [8]. In a clinical study, Tregerman and coworkers treated nine patients with each of the three frameworks differing in manufacturing and impression technique. With the classic impression technique and physical models, casting performed slightly better than SLM. However, the complete digital workflow, intraoral scanning, and SLM performed best [28].

**Figure 11:**
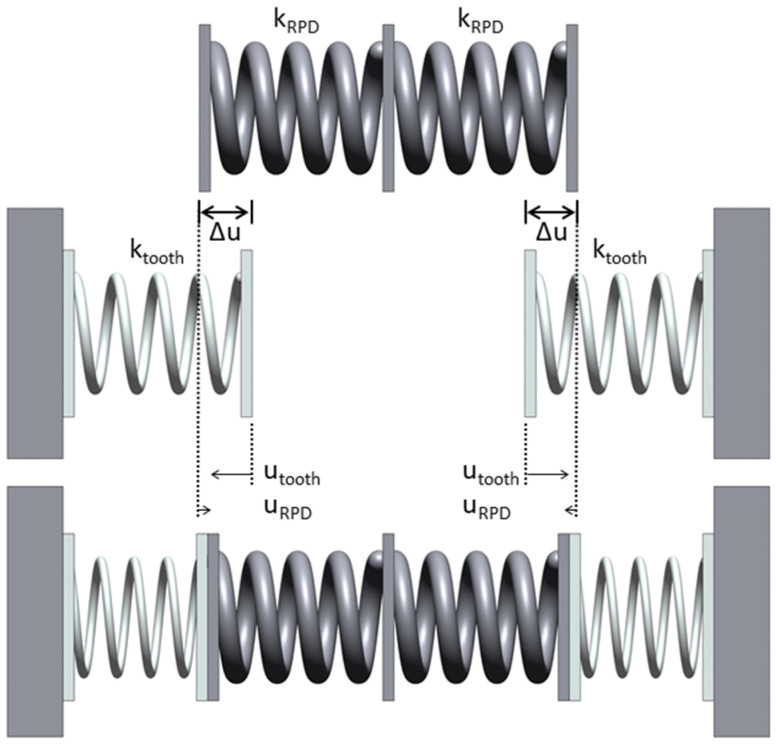
Mechanical model to describe deflection of teeth (*u_tooth_*) and RPD (*u_RPD_*) due to RPD insertion with a misfit Δ*u*. The top and center rows show the stress-free states before insertion. The bottom row shows the state with inserted RPD. The misfit Δ*u* will be split up into tooth and RPD deflections based on the stiffnesses of the teeth (*k_tooth_*) and RPD (*k_RPD_*) with the more resilient (less stiff) element showing the larger deflection.

It must be discussed that displacements of the teeth (proportional to forces acting on the teeth) are smaller than the manufacturing inaccuracies of the RPDs since RPD stiffness (*k_RPD_*) is not very much larger than the stiffness of the teeth (*k_tooth_* = 100 N/0.39 mm) in our model. Based on simple FE models, we estimated the stiffness in the anterior direction between two clasp elements to the *k_RPD,anterior_* > 4 × *k_tooth_* and *k_RPD,lateral_* = 2 × *k_tooth_* for all design types. With the simple model shown in Figure 11, it can be derived that the tooth deflection for a misfit Δ*u* in the observed direction is given by $u_{tooth}=\left(k_{RPD}\cdot \Delta u\right)/\left(k_{RPD}+k_{tooth}\right)$. 

Hence, in a lateral direction, tooth deflection will be about 67% of the misfit whereas, in the anterior direction, tooth deflection is >80% of the misfit, and, consequently, the real misfit (measured at one element) will be up to factor 1.5 larger than the tooth deflection correlating with the measured force. Often misfit is measured with a caliper between two opposing elements. For such a measurement, the resulting misfit would be the sum of both single discrepancies, i.e., twice as high when using the averaged values presented here. Exemplarily, the deflection of opposing teeth of 15 µm each would correspond to an overall inaccuracy (distance deviation) of the RPD of 40 to 45 µm. This behavior is not necessarily a flaw of our method since it is caused by the mechanical properties of the RPD and will be the same when inserted inside the patient’s mouth.

Besides our investigation finding clinically acceptable initial retention, the retention of SLM-fabricated clasps in comparison to cast counterparts has been the subject of several studies. One study found no significant difference in the initial retentive force between SLM cobalt–chromium and cast clasps. However, after an aging simulation with 7200 insertion/removal cycles, the SLM clasp exhibited a greater residual retentive force [29]. This suggests that the SLM clasps maintained its retention better over time compared to the cast clasps. In another study comparing SLM titanium clasps to cast titanium clasps, it was found that SLM clasps had better fatigue resistance. The decrease in retentive force for SLM clasps was less than that of the cast group after a certain number of insertion/removal cycles [30]. This also indicates that SLM clasps may retain their effectiveness for a longer period. Schweiger at al. found initial retentive forces of 13.6 N (cast) and 15.7 N (SLM) for frameworks [18]. Interestingly, the values declined significantly with aging in the cast group but not in the SLM group, which indicates that retention is not problematic with SLM fabricated RPDs. However, contrasting results were observed in a study by Tan et al., which focused on SLM titanium clasps. Initially, the SLM titanium clasp exhibited significantly higher retentive forces than the other groups. However, after 2000 insertion/removal cycles, the retentive forces rapidly reduced [31]. This suggests that while SLM titanium clasps may initially provide strong retention, their long-term performance may be compromised. Overall, the retention of SLM clasps compared to cast clasps is still a topic of investigation, and the findings vary depending on the specific material and design of the clasp, as well as the testing conditions. Further research, as performed in our investigation, is needed to fully understand the retention capabilities of SLM clasps.

A shortcoming of our study was that aging could not be simulated. Since we worked with a single master model providing natural teeth, this model must not suffer from wear due to repetitive insertion and removal of the RPDs corresponding to several years of use by a patient.

In addition to the two main study limitations stated above, several further aspects require discussion. First, even though the frameworks were designed to reflect typical clinical configurations, the study only tested three of the many different available types of abutment configurations, framework designs, and clasps. Second, no optimization of SLM parameters was performed. The used parameters were recommended by the supplier based on their best knowledge and clinical experience. Third, we tried to closely simulate the oral cavity by using a biomechanical model with extracted teeth and tooth-like resilience. Nonetheless, this model had limitations; namely, vertical misfits were not assessed at all. Future clinical studies need to include vertical deviations and other patient-related outcomes. For a given misfit, the magnitude of *F_res_* depends on tooth resilience, which was 0.39 mm per 100 N in this model. It should, therefore, be kept in mind that forces might differ if a patient’s tooth resilience differs from this value. Especially if abutment teeth are periodontally compromised, resilience will increase, and misfit-induced constraint forces will decrease. Lastly, no conclusions can be drawn regarding SLM material fatigue and wear. The dentures were fitted and removed six times only. Improvements in post-processing might further increase mechanical properties [10] and reduce anisotropic corrosion behavior [32] but were beyond the scope of this investigation.

The average horizontal constraint forces were below 7 N, and the maximum horizontal constraint forces never exceeded 18 N for all designs and manufacturing techniques. These values lie in a clinically acceptable range.

When looking at the microstructure of the RPDs fabricated with either casting or SLM, SLM-fabricated RPDs showed a more homogeneous microstructure and fewer flaws. This is an advantage of the SLM technique.

## 5. Conclusions

Besides the lost-wax casting technique clinically used in dentistry for a long time to provide patients with clasp-retained RPDs, new techniques such as SLM now provide alternative, digital fabrication possibilities.

This study compared clasp-retained RPDs fabricated using both SLM and casting techniques. No significant difference was found with regard to average misfit-induced forces between the two methods. Both fabrication methods exhibited mean inaccuracies of the RPDs below 40 µm, highlighting the challenges in achieving a perfectly passive fit. Nevertheless, both SLM and casting provided clinically acceptable fit. SLM-fabricated RPD frameworks demonstrated slightly higher retention forces compared to cast RPDs, meeting the specified target values for a single abutment tooth between 5 and 10 N. Based on these results, SLM-fabrication seems to be a viable alternative technology to fabricate RPDs with good clinical performance. It is important to note that further research is needed to investigate other factors such as vertical deviations and long-term clinical performance.

## Data Availability

The data that support the findings of this study are available from the corresponding author, S.R., upon reasonable request.
